# 

**Published:** 1904-07

**Authors:** 


### TEXAS Medical Journal.

Subscription, $1.00 a Year, in Advance.

Single Copies, 10 Cents.

PUBLISHED AT AUSTIN, TEXAS, BY
F. E. DANIEL, M. D.,
Proprietor.

PRINTED BY THE VON BOECKMANN-JONES CO.

Entered at the Postoffice at Austin, Texas, as Second-class Mail Matter.

